# Lexical processing of Chinese sub-character components: Semantic activation of phonetic radicals as revealed by the Stroop effect

**DOI:** 10.1038/s41598-017-15536-w

**Published:** 2017-11-17

**Authors:** Su-Ling Yeh, Wei-Lun Chou, Pokuan Ho

**Affiliations:** 1 Department of Psychology, National Taiwan University, Taipei, Taiwan; 2 Graduate Institute of Brain and Mind Sciences, National Taiwan University, Taipei, Taiwan; 3 Neurobiology and Cognitive Science Center, National Taiwan University, Taipei, Taiwan; 4 Department of Psychology, Fo Guang University, Yilan, Taiwan

## Abstract

Most Chinese characters are compounds consisting of a semantic radical indicating semantic category and a phonetic radical cuing the pronunciation of the character. Controversy surrounds whether radicals also go through the same lexical processing as characters and, critically, whether phonetic radicals involve semantic activation since they can also be characters when standing alone. Here we examined these issues using the Stroop task whereby participants responded to the ink color of the character. The key finding was that Stroop effects were found when the character itself had a meaning unrelated to color, but contained a color name phonetic radical (e.g.,  “guess”, with the phonetic radical  “cyan”, on the right) or had a meaning associated with color (e.g.,  “pity”, with the phonetic radical  “blood” on the right which has a meaning related to “red”). Such Stroop effects from the phonetic radical within a character unrelated to color support that Chinese character recognition involves decomposition of characters into their constituent radicals; with each of their meanings including phonetic radicals activated independently, even though it would inevitably interfere with that of the whole character. Compared with the morphological decomposition in English whereby the semantics of the morphemes are not necessarily activated, the unavoidable semantic activation of phonetic radicals represents a unique feature in Chinese character processing.

## Introduction

Unlike alphabetic writing systems (e.g., English) where their grapheme-to-phoneme (letter-to-sound) correspondence rules play an important role in reading, Chinese’s ideographic nature and their lack of such correspondence rules make them an excellent tool for providing key comparisons with alphabetic systems. Some researchers believed that by illuminating the common and unique aspects and examining central theoretical issues in reading between Chinese and alphabetic systems such as how morphemic units are processed, it is possible to develop general models for reading and specify the constraints imposed by different writing systems^1^.

**Figure 1:**
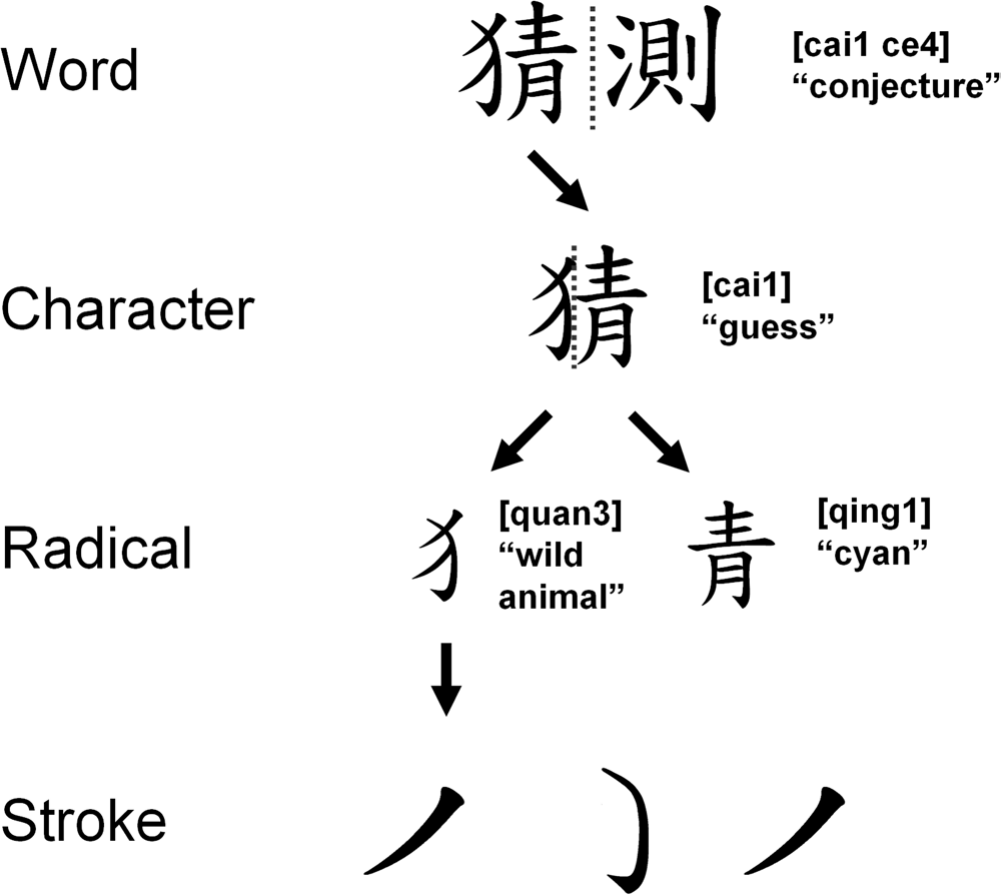
The four levels of Chinese word complexity: Word, Character, Radical, and Stroke. Content inside square-brackets denote pronunciations.

In Chinese script, there are four levels of structural complexity to a Chinese word: *stroke*, *radical*, *character*, and *word* (Fig. 1). *Stroke* is the smallest structural unit that form a single character but single strokes generally do not have meaning. *Radicals* are simple meaningful linguistic units that are each formed by a group of strokes and are building blocks of a single character. *Characters* are single functional mono-syllabic logograms that may be made up by two or more radicals, in which cases are called “compound characters”. However, the distinction between radicals and characters is not always clear-cut, as the same group of strokes could be considered a radical when it is combined with other radicals, or a character when it stands alone in many cases. A Chinese *word* is a syntactic unit governed by sentence structure rules and can be categorized into one or more syntactic categories such as verbs, nouns, adjectives, etc.; lengths of Chinese words could range from one to multiple characters.

Most compound characters (70–80%) are systematically constructed by a semantic radical and a phonetic radical. A semantic radical is a categorical unit of meaning that can be used to index the character in a Chinese dictionary, and the phonetic radical usually provides the phonological clue for character pronunciation. In most cases, these radicals can also be classified as characters when they are isolated. For example, the character  (pronounced as [mei3], meaning “magnesium”; the number within the brackets denotes the lexical tone of the syllable) is a compound character that includes the semantic radical,  ([jin1], “metal”), which indicates semantic relation to metal, and the phonetic radical,  ([mei3], “beautiful”), which cues the pronunciation of the character. Although the cueing validity of the two radical types varies from low to high among the population of Chinese characters and is about 30~40% on average^2,3^, past studies have suggested that semantic and phonetic radicals may still provide useful information about the meaning and the pronunciation of the characters, respectively^4–7^.

When it comes to the investigation of phonetic radicals’ roles, past studies have mainly focused on, and indeed found, phonological activation of phonetic radicals and its effect on naming the characters in which they are embedded^7–13^. However, to fully understand the extent of radical processing, it is imperative to examine whether phonetic radicals’ meanings also become activated, in addition to that from semantic radicals. In theory, the semantic activation of a phonetic radical should conflict with the whole character it is embedded in, since functionally its role should only be providing pronunciation cues. Take the aforementioned character  for example, the phonetic radical  (pronounced as [mei3]) can stand alone as a character with the meaning “beautiful”, which is distinct from the meaning of the compound character  ([mei3], “magnesium”), but little is known about whether  is nevertheless semantically activated. Hence, in the current study, we ask specifically whether the *meaning* of the phonetic radical, which supposedly only cues the sound of the compound character, is also activated during character recognition. If so, combined with the known evidence from findings of semantic activation of semantic radicals, this might argue for the proposition that all Chinese radicals, regardless of their type (semantic or phonetic), go through the same lexical process and semantic activation as whole characters.

Given the importance of this research question, it is quite surprising to find that only few relevant empirical results exist, which suggest a positive answer. For example, Zhou and Marslen-Wilson^13^ (Experiment 2) found that a prime character (e.g., , [po1], ”drift”, containing a phonetic radical , [bai2], “white”) could facilitate the naming latency of a target character (e.g., , [hei1], meaning “black”). However, since the observed magnitude of this priming effect was minuscule (9.5 ms on average from about 100 participants), this finding has, in fact, evoked doubts among researchers in this field. The gist of such doubts is well captured in Liu’s previous work^14^, which pointed out that it would be too remarkable if Chinese readers could accomplish such great deal of work that “under some circumstances read two thousand additional words simultaneously when reading a one thousand word corpus”. Thus, while the conclusion that radical processing affects character recognition is relatively uncontroversial, whether phonetic radicals are semantically processed remains an unresolved debate.

In order to provide an unequivocal test, it is desirable to approach from a different paradigm which may give rise to a more robust effect. The color-naming Stroop task^15^ is one such paradigm, which can address the possibility of semantic activation of the phonetic radical. In a typical Stroop color-naming task, the participants are asked to name the ink color of the target item. Previous studies using English words as stimulus materials have demonstrated that naming the color of an incongruent color word (e.g., *blue* written in red) was slower than naming the color of a neutral word unrelated to color. Also commonly observed is a facilitation effect in which naming the color of a congruent word (e.g., *blue* written in blue) is faster than naming the color of the neutral control, though this effect is usually not as robust or consistent as the interference effect^16^. The interference and facilitation effects have been taken as evidence for unavoidable word processing up to semantic level^17,18^ and can thus be exploited to investigate the question of whether phonetic radicals are also semantically processed in a compound character.

In the current study, we used the Stroop paradigm to examine whether a phonetic radical’s meaning is also activated in a character. Previous studies have used the Stroop paradigm and found semantic activation of radicals^19,20^, but they did not differentiate semantic vs. phonetic radicals, and especially did not emphasize on the possibility of semantic activation of phonetic radicals. Semantic radicals can activate their meanings as shown in previous studies^4–6^, hence if the radicals being investigated included semantic radicals, their meanings may have more likely been activated and led to the Stroop effect. The current study particularly emphasized on the semantic activation of phonetic radicals; if it can be proven that even phonetic radicals’ semantics are activated in reading, it would be a strong support for the notion that radical’s processing is vastly similar to character’s. Furthermore, in this study we differentiated whether the phonetic radical was a valid (regular) or invalid (irregular) one in terms of cuing the pronunciation of the character, and if there is no difference between the two conditions under the Stroop paradigm, it implies semantic activation carry more weight than sound which may also shed light on phonetic radical processing in reading Chinese characters.

To provide an overview of our study, four experiments using variations of the Stroop color-naming task were conducted, in which the critical stimuli relied on the use of characters containing phonetic radicals that are color names or objects associated with color, but have conflicting pronunciations and meanings from the compound characters they appeared in. In summary, in Experiment 1, we investigated the Stroop effects (both facilitation and interference) for three character types to survey the strengths of the Stroop effects we should expect to observe. In Experiment 2, we focused only on the critical stimuli to avoid priming from trials in other conditions. In Experiment 3, we presented the critical stimuli in multiple character presentations to roughly simulate the effect in a more-normal reading condition; a reading comprehension task was also given unexpectedly after the color naming task. And in Experiment 4, on top of the critical stimuli, we added a new character type in which the containing phonetic radicals are not color names in meaning nor pronunciation, and only shares semantic associations with color (i.e., colorful objects such as “blood” which has association with “red”).

## Methods

### Approval, Accordance, and Informed Consent

The studies were approved by the ethic committee in the Department of Psychology at National Taiwan University. The experiments were conducted in accordance with applicable research subject guidelines. All participants gave informed consent prior to data collection.

### Experiment 1 – Examining Stroop effects for all character types

#### Participants

All experiments recruited native Chinese speaking undergraduate students of National Taiwan University. All participants had normal or corrected-to-normal vision and were naïve to the purpose of the experiments; they were also rewarded with a small fee for their contribution. Thirty students participated in Experiment 1.

#### Stimuli and Design

Stimuli were displayed on a 15-inch CRT monitor and controlled by a Pentium III personal computer (refresh rate: 52 Hz) using DMDX^21^ with a gray background color (RGB: 150, 150, 150). The characters were printed in the Kai font () subtended at a visual angle of 1.5° (width) × 1.7° (height), and presented in one of the three colors: red (RGB: 255, 0, 0), yellow (RGB: 255, 255, 0), or cyan (RGB: 0, 255, 255). The color patch was a rectangle having the same size as the character, and filled with one of the three colors (red, yellow, or cyan). A gray disk (0.95° in diameter; RGB: 128, 128, 128) was centered on the screen as the fixation point.

Participants were tested individually, sitting at a viewing distance of 60 cm from the computer screen in a quiet experimental chamber. They were told to read the color of the presented character in each trial into a microphone, and the voice onset time was recorded by the computer connected to the microphone. The experimenter recorded the participant’s responses and compared them with the correct answers after the experiment.

There were three character types: the *Color-Character*, *Valid-Radical*, and *Invalid-Radical* conditions. The *Color-Character* condition consists of simple characters in which the meaning of each word directly refers to a color (e.g., , [qing1], “cyan”), as typically used in the conventional Stroop task. The *Valid-Radical* condition is made up of characters that share the same pronunciations with their embedded phonetic radicals but with dissimilar meanings (e.g., , [qing1], “clear” is pronounced the same way as , [qing1], “cyan”). The *Invalid-Radical* condition contains characters that share neither meanings nor pronunciations with their respective phonetic radicals (e.g., , [cai1], “guess” is both semantically and phonetically distinct from , [qing1], “cyan”). Additionally, for each character we selected a *Neutral-Control* character matched in both usage frequency and stroke count. The selection criterion was that neither character nor its radical has a pronunciation or meaning related to color names. Table 1 gives an example of each of the three conditions and their respective *Neutral-Controls*. See supplemental material 1.1 for a list of all characters used and their characteristics at the radical level. As it is already quite difficult to find these well controlled stimuli, we did not control consistency and neighborhood size. Nevertheless, we compared the results of *Valid-* vs. *Invalid*-*Radicals* with the *Neutral-Control* characters across different conditions. As identical radicals were used in these two conditions, the regularity, consistency, and neighborhood size should be the same for the two radical conditions, hence any difference in the Stroop effects obtained should not be attributed to these factors.

**Table 1:** Examples of Chinese characters used in this study.

Condition	*Color-Character*	*Color-Character*’*s Neutral-Control*	*Valid-Radical*	*Valid-Radical*’*s Neutral-Control*	*Invalid-Radical*	*Invalid-Radical*’*s Neutral-Control*	*Associative-Radical*	*Associative-Radical*’*s Neutral-Control*
Character								
Meaning	cyan	tool	clear	reason	guess	tent	pity	occupy
Pronunciation	[qing1]	[ju4]	[qing1]	[li3]	[cai1]	[zhang4]	[xu4]	[zhan4]
Phonetic radical	—	—						
Meaning	—	—	cyan	length unit	cyan	long	blood	divine
Pronunciation	—	—	[qing1]	[li3]	[qing1]	[chang2]	[xie3]	[zhan1]

Note that the *Color-Character*, *Valid-Radical*, *Invalid-Radical* and their *Neutral-Controls* were used in Experiment 1. Only the *Invalid-Radical* condition and its *Neutral-Controls* were used in Experiment 2 and 3. The *Associative-Radical* condition and its *Neutral-Controls* were used in Experiment 4.

In total, three characters were chosen for each of the three conditions and their controls, yielding a sum of 18 characters used in this experiment: 3 conditions × (3 characters + 3 controls). For each of the three conditions (*Color-Character*, *Valid-Radical*, and *Invalid-Radical*), two kinds of trials were constructed: congruent and incongruent trials. In the congruent trials, Chinese characters were shown in colors consistent with the meanings of the whole characters in the *Color-Character* condition, and with the meanings of the phonetic radicals in the *Valid-Radical* and *Invalid-Radical* conditions. In the incongruent trials, these characters were shown in colors inconsistent with the meanings of both the whole characters in the *Color-Character* condition and the phonetic radicals in the *Valid-Radical* and *Invalid-Radical* conditions. Matched *Neutral-Control* characters were also presented in these trials to provide a baseline to assess the Stroop effect.

There were 120 experimental trials divided into two blocks of 60 trials each. Within each block, there were 54 character trials [3 conditions × (3 characters + 3 controls) × 3 colors] and 6 color patch trials. Each block consisted of 9 congruent trials (3 characters × 1 color × 3 conditions, excluding the *Neutral-Control* characters), 18 incongruent trials (3 characters × 2 colors × 3 conditions, excluding the *Neutral-Control* characters), 27 *Neutral-Control* trials (3 *Neutral-Control* characters × 3 colors × 3 conditions), and 6 color patch trials (3 colors × 2 trials). For all experiments in this study, the incongruent trials used all possible combinations of characters and colors except for the congruent combinations. All trials were presented in a completely randomized order.

#### Procedure

Participants initiated the first trial of each block by pressing the space bar. At the start of each trial, the fixation disk was shown for 347 ms, followed by the target character at the same location, waiting for the participant to respond. The participants were asked, while ignoring the identity of each character, to name the color of the character as quickly and accurately as possible. The naming latency was defined as the time between the stimulus onset and the response collected from the voice key. After the response, a feedback tone presented for 50 ms was given to inform the participant that the voice key had received the signal. A trial without the feedback tone would be coded as a voice-key error and would be excluded from later analyses. Twenty-four practice trials divided equally between the three colors preceded the experimental trials. In all the experiments reported in this study, the stimuli used in the practice blocks were not presented in the experimental blocks.

### Experiment 2 – Invalid-Radical, the critical character type

#### Participants

Twenty-six students participated.

#### Stimuli, Design, and Procedure

The stimuli, design, and procedure were the same as in Experiment 1, except that now only the *Invalid-Radical* condition was used, excluding the *Color-Character* and *Valid-Radical* conditions. The 96 experimental trials in total were divided into two blocks of 48 trials each. Within each block, there were 36 character trials and 12 color patch trials (3 colors × 4 trials). The character trials consisted of 6 congruent trials (3 characters × 1 color × 2 trials, excluding the *Neutral-Control* characters), 12 incongruent trials (3 characters × 2 colors × 2 trials, excluding the *Neutral-Control* characters), and 18 *Neutral-Control* character trials (3 characters × 3 colors × 2 trials).

### Experiment 3 – Invalid-Radical during reading

#### Participants

Seventeen students participated.

#### Stimuli, Design, and Procedure

The stimuli, design, and procedure were identical to our previous experiments, except for the following: the stimuli went from single characters to four-character phrases subtended at a visual angle of 6° (width) × 1.7° (height) (each character: 1.5° × 1.7°). For each phrase, three initial characters were in black, and the last one was in the specified color (red, yellow, or cyan). To give an example, one of our chosen phrases were  (Meaning: describes childhood innocence, Pronunciation: [liang3 xiao3 wu2 cai1]); the critical character  was presented in one of the three colors while the first three characters  were drawn in black. To account for possible task response strategies (i.e., focus only at where the colored character would appear), we implemented two changes. First, we split the screen into four quadrants where the fixation disk and the four-character stimulus would appear with their respective timing in one of the four quadrants within the same trial. The fixation disk’s location within the quadrant was the same as the first character (left-most) of the four-character phrase to encourage reading from the phrase’s starting position, and the four-character phrase is center-aligned within the quadrant. The order for the different quadrants was pseudorandomized to ensure that all quadrants had the same number of trials. Second, we removed the color patch trials for this experiment because a single color patch could not be integrated with the first three characters from a four-character phrase to form a meaningful message, and displaying just one patch similar to previous experiments would produce more complications such as introducing inconsistent stimulus sizes.

To make this experiment comparable to our Experiment 2, the critical characters of our matched neutral phrases were the same characters as the matched control in Experiment 2. The participants were asked to name the ink color of the colored character which could have been one of the *Invalid-Radical* characters or the *Neutral-Control* characters. In congruent trials, these colored characters were drawn in colors consistent with the meanings of the phonetic radicals; in incongruent trials, they were drawn in colors different from the meaning of their phonetic radical. In each condition, three four-character phrases were chosen as stimuli (see supplemental material 1.3 for a complete list of all phrases used). Moreover, in order to check whether the participants read the phrases, the participants were given a phrase recognition task after the main experiment.

There were 36 experimental trials which consisted of 6 congruent trials (3 characters × 1 color × 2 phrases, excluding the *Neutral-Control* characters), 12 incongruent trials (3 characters × 2 colors × 2 phrases, excluding the *Neutral-Control* characters), and 18 *Neutral-Control* trials (3 characters × 3 colors × 2 phrases). The phrase recognition task was made up of six two-phrase pairs as forced-choice questions and the participants were asked to choose one phrase which was presented in the previous color naming task from each pair. The two phrases of each force-choice pair contained the same critical character but had different remaining characters to prevent participants from reliably choosing the correct answer based on the critical character alone.

### Experiment 4 – Associative-Radical

#### Participants

Thirty students participated.

#### Stimuli, Design, and Procedure

We limited our scope to only the Stroop interference effect. On top of the *Invalid-Radical* condition, we also added the *Associative-Radical* condition made up of characters that contained phonetic radicals that are not color names in meaning nor pronunciation, but are nonetheless semantically associated with color (e.g., , [xu4], “pity” with the phonetic radical , [xie3], “blood”, which is semantically associated with the color “red”). As with the *Invalid-Radical* condition, *Neutral-Control* characters matched in usage frequencies and stroke counts were paired with each *Associative-Radical* character. There were 120 experimental trials, divided into two blocks of 60 trials each. Within each block, there were 48 character trials and 12 color patch trials. Each block consisted of 24 incongruent trials (2 conditions × 3 characters × 2 colors × 2 trials, excluding the *Neutral-Control* characters), 24 *Neutral-Control* character trials (2 conditions × 3 characters × 2 colors × 2 trials), and 12 color patch trials (3 colors × 4 trials). Twenty-four practice trials preceded the experimental blocks. Other details were the same as in Experiment 1.

### Statistical Analysis

To carry out the Linear Mixed Effect (LME) Model^22^ analysis, we incorporated the ‘lme4’^23^ (ver. 1.1–13), the ‘lsmeans’^24,25^ (ver. 2.26–3), and the ‘RePsychLing’^26^ (ver. 0.0.4) packages from the statistical analysis software R (ver. 3.4.1). All four experiments share the same general procedure; however, since Experiment 2 and 3 had one less fixed factor (only 1 character type: *Invalid-Radicals*) than Experiment 1 and 4 (multiple character types), we removed “character type” as a factor in Experiment 2 and 3. As recommended by Bates and colleagues^26^, we analyzed our data based on a parsimonious version of the “Most-Maximal-Possible-Model” (MMP-Model), which is one with the most complex random effect structure that converges without warning or error. We will briefly outline the general procedure below, but we encourage readers to consult Supplemental Material 2 for more details of each step and codes of our R implementation; additionally, line-by-line explanations of our R commands are also available from the link we provide in the Data Availability Statement section.

Our analysis comprises of 3 steps: (1) Determining the MMP-Model using ‘lme4’. (2) Reduce the found model systematically to avoid over-specification using ‘RePsychLing’. (3) Construct comparison tables using ‘lsmeans’ with the final model from step 2 as a parameter. For the comparisons, we employed Dunnett’s method for the comparisons of “*Congruent* vs. *Neutral-Control*” and “*Incongruent* vs. *Neutral-Control*” within each Character Type; furthermore, a Holm-Bonferroni Correction^27^ was applied wherever an exploratory comparison was conducted. As recommended by Streiner^28^, we will include both corrected and uncorrected p-values for significant results but base our conclusions on the corrected p-values.

## Results

**Figure 2:**
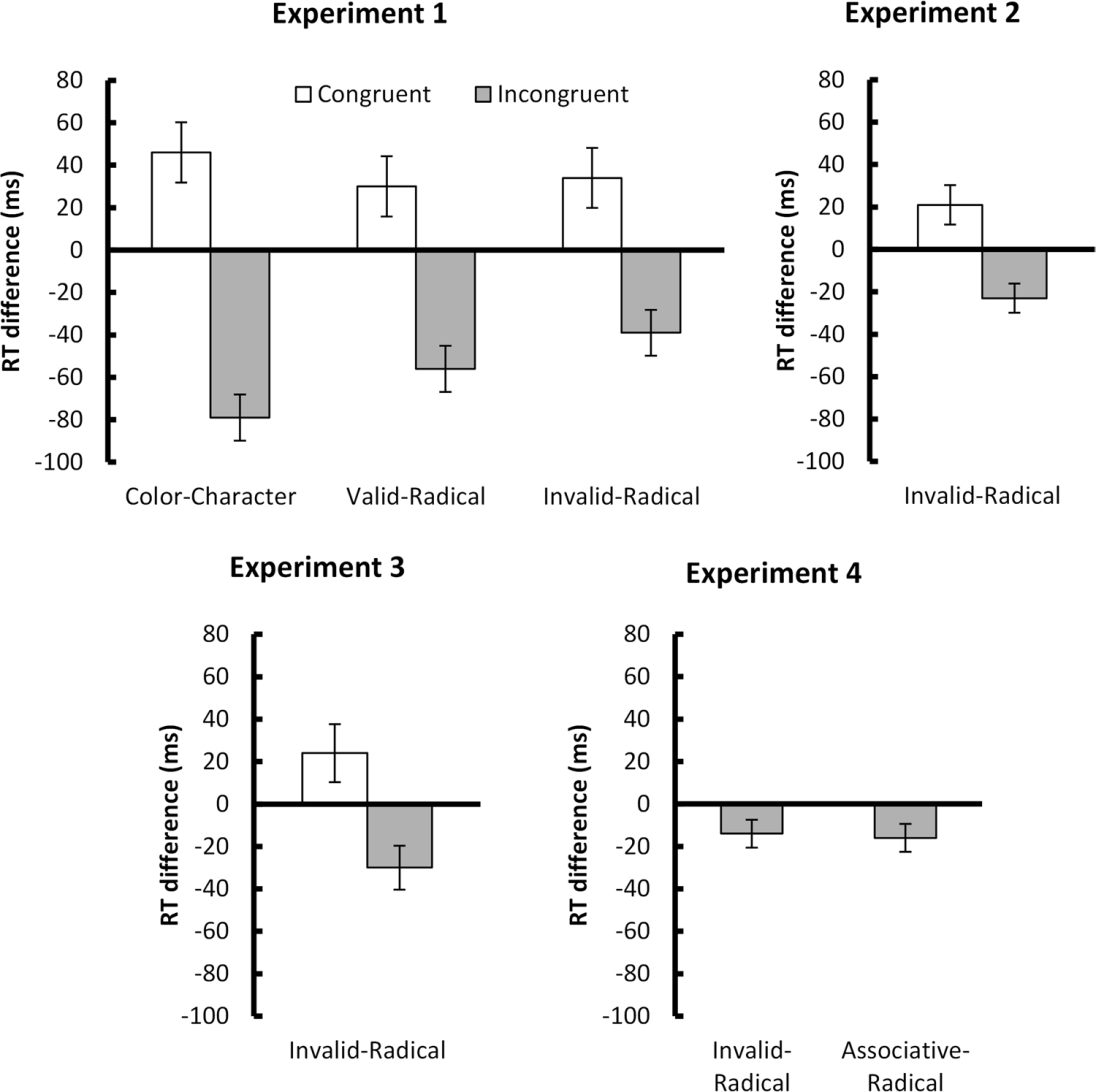
Results of this study. RT differences = RT (*Neutral-Control* trials) - RT (non- *Neutral-Control* trials). Positive values denote RT facilitation (faster than RT from *Neutral-Controls*), and negative values indicate RT interference (slower than RT from *Neutral-Controls*). Error bars represent one standard error from the mean.

Figure 2 shows the graphical representation of the RT differences in each condition for all four Experiments. Table 2 summarizes the descriptive statistics.

**Table 2:** Mean reaction times (RT, ms), error rates (ER, %), and standard errors (SE, in parentheses) as a function of congruency and character type from the LME model.

	*Experiment 1*				*Experiment 2*	
		*Color-Character*	*Valid-Radical*	*Invalid-Radical*		*Invalid-Radical*
*Congruent*	RT (SE)	582.63 (27.39)	600.59 (27.36)	579.92 (27.36)	RT (SE)	429.17 (21.08)
	ER (SE)	5.36 (2.15)	4.17 (2.15)	2.98 (2.15)	ER (SE)	4.49 (1.81)
*Incongruent*	RT (SE)	707.30 (25.82)	686.68 (25.82)	652.48 (25.82)	RT (SE)	473.56 (20.09)
	ER (SE)	11.61 (1.76)	7.74 (1.76)	7.74 (1.76)	ER (SE)	7.69 (1.57)
*Control*	RT (SE)	628.30 (25.29)	630.67 (25.28)	613.63 (25.28)	RT (SE)	450.10 (19.75)
	ER (SE)	4.96 (1.61)	3.57 (1.61)	3.97 (1.61)	ER (SE)	4.59 (1.48)
	***Experiment 3***		***Experiment 4***			
		***Invalid-Radical***		***Invalid-Radical***	***Associative-Radical***	
*Congruent*	RT (SE)	482.88 (24.30)	RT (SE)	—	—	
	ER (SE)	2.94 (3.22)	ER (SE)	—	—	
*Incongruent*	RT (SE)	536.89 (22.61)	RT (SE)	579.53 (17.74)	584.05 (17.73)	
	ER (SE)	10.78 (2.72)	ER (SE)	8.61 (3.34)	9.17 (3.29)	
*Control*	RT (SE)	507.12 (22.03)	RT (SE)	565.78 (17.35)	567.57 (17.26)	
	ER (SE)	7.19 (2.53)	ER (SE)	6.94 (3.25)	6.94 (3.20)	

### Experiment 1 – Examining Stroop effects for all character types

Two out of 30 participants were removed due to unexpected technical difficulties. For the rest of the data, naming latencies above 1200 ms and below 200 ms were excluded. The removal rate was lower than 1%. The Parsimonious Model had the formula:

$$\begin{array}{rcl} > \mathrm{lmer}\_\mathrm{object} & = & {\rm{lmer}}({\rm{RT}} \sim {\rm{congruence}}\ast \mathrm{character}\_\mathrm{type}+(1|\mathrm{subject})\\  &  & +(1|\mathrm{pair})+(1|\mathrm{color}),{\rm{data}}={\rm{stroop}}{\rm{.data}})\end{array}$$

Planned comparisons showed that, relative to *Neutral-Control* trials, significant facilitation effects were found in the *Color-Character* (M = 46 ms, SE = 14.2 ms, *t* (369.84) = 3.214, *uncorrected p* = 0.0014, *corrected p* = 0.0028, *d* = 0.35) and *Invalid-Radical* (M = 34 ms, SE = 14.1 ms, *t* (360.56) = 2.384, *uncorrected p* = 0.0177, *corrected p* = 0.0339, *d* = 0.26) condition. Marginal facilitation effect was found in the *Valid-Radical* (M = 30 ms, SE = 14.1 ms, *t* (362.79) = 2.127, *uncorrected p* = 0.0341, *corrected p* = 0.0644, *d* = 0.23) condition. Interference effects were also found in all three conditions (*Color-Character* condition: M = 79 ms, SE = 10.9 ms, *t* (1003.38) = 7.244, *uncorrected p* < 0.0001, *corrected p* < 0.0001, *d* = 0.56; *Valid-Radical* condition: M = 56 ms, SE = 10.9 ms, *t* (1008.84) = 5.141, *uncorrected p* < 0.0001, *corrected p* < 0.0001, *d* = 0.40; *Invalid-Radical* condition: M = 39 ms, SE = 10.9 ms, *t* (1013.83) = 3.570, *uncorrected p* = 0.0004, *corrected p* = 0.0007, *d* = 0.28). Further analysis showed that interference effects were stronger in the *Color-Character* condition (79 ms) than in the *Invalid-Radical* condition (39 ms; difference = 40 ms, SE = 15.4 ms, *t* (1008.54) = 2.606, *uncorrected p* = 0.0093, *corrected p* = 0.0279, *d* = 0.20), but no other meaningful differences were found.

LME analysis of error rates indicated that there was no speed-accuracy trade-off. While error rates were similar between congruent trials and *Neutral-Control* trials, error rates were higher for incongruent trials in all conditions relative to *Neutral-Control* trials (*Color-Character* condition: M = 6.6%, SE = 1.6%, *t* (1474) = 4.146 *uncorrected p* < 0.0001, *corrected p* = 0.0001; *Valid-Radical* condition: M = 4.2%, SE = 1.6%, *t* (1474) = 2.599, *uncorrected p* = 0.0094, *corrected p* = 0.0183; *Invalid-Radical* condition: M = 3.8%, SE = 1.6%, *t* (1474) = 2.352, *uncorrected p* = 0.0188, *corrected p* = 0.0361). No other effects were found.

Going back to our choice of the parsimonious model, even though the one we arrived at did not significantly differ with the MMP-Model, there was a trend toward marginal significance (*p* = 0.121), and thus one might wonder whether our results would change had we adopted the more complex model. The MMP-Model had the formula:

$$\begin{array}{rcl} > \mathrm{lmer}\_\mathrm{object} & = & \mathrm{lmer}(\mathrm{RT} \sim {\rm{congruence}}\ast \mathrm{character}\_\mathrm{type}+(1+\mathrm{congruence}|\mathrm{subject})\\  &  & +(1|\mathrm{pair})+(1|\mathrm{color}),{\rm{data}}={\rm{stroop}}{\rm{.data}})\end{array}$$

Analysis showed there was no decisional difference in facilitation effects (*Color-Character* condition: M = 46 ms, SE = 14.2 ms, *t* (351.48) = 3.214, *uncorrected p* = 0.0014, *corrected p* = 0.0028, *d* = 0.35; *Valid-Radical* condition: M = 30 ms, SE = 14.2 ms, *t* (344.86) = 2.124, *uncorrected p* = 0.0344, *corrected p* = 0.0648, *d* = 0.23; *Invalid-Radical* condition: M = 34 ms, SE = 14.2 ms, *t* (342.88) = 2.379, *uncorrected p* = 0.0179, *corrected p* = 0.0343, *d* = 0.26), interference effects (*Color-Character* condition: M = 79 ms, SE = 11.3 ms, *t* (373.82) = 7.020, *uncorrected p* < 0.0001, *corrected p* < 0.0001, *d* = 0.54; *Valid-Radical* condition: M = 56 ms, SE = 11.3 ms, *t* (373.58) = 4.980, *uncorrected p* < 0.0001, *corrected p* < 0.0001, *d* = 0.38; *Invalid-Radical* condition: M = 39 ms, SE = 11.3 ms, *t* (373.29) = 3.466, *uncorrected p* = 0.0006, *corrected p* = 0.0012, *d* = 0.27), and how they compared across conditions (significant difference between *Color-Character* and *Invalid-Radical* conditions’ interference effect: difference = 40 ms, SE = 15.4 ms, *t* (1009.51) = 2.613, *uncorrected p* = 0.0091, *corrected p* = 0.0273, *d* = 0.20).

### Experiment 2 – Invalid-Radical, the critical character type

Same trimming procedure as Experiment 1 was applied. Trial removal rate was lower than 1%. The Parsimonious Model had the formula:

$$\begin{array}{rcl} > \mathrm{lmer}\_\mathrm{object} & = & \mathrm{lmer}(\mathrm{RT} \sim {\rm{congruence}}+(1|\mathrm{subject})\\  &  & +(1|\mathrm{pair})+(1|\mathrm{color}),{\rm{data}}={\rm{stroop}}{\rm{.data}})\end{array}$$

Relative to *Neutral-Control* trials, *Invalid-Radical* condition yielded a Stroop facilitation effect (M = 21 ms, SE = 9.4 ms, *t* (236.64) = 2.236, *uncorrected p* = 0.0263, *corrected p* = 0.0500, *d* = 0.25) and an interference effect (M = 23 ms, SE = 6.9 ms, *t* (385.71) = 3.417, *uncorrected p* = 0.0007, *corrected p* = 0.0014, *d* = 0.39).

Analysis of error rates indicate that the speed-accuracy trade-off can be ruled out. Incongruent *Invalid-Radical* characters produced more naming errors than *Neutral-Control* characters (M = 3.1%, SE = 1.2%, *t* (275.80) = 2.687; *uncorrected p* = 0.0077, *corrected p* = 0.0149). There were no other effects of naming errors.

### Experiment 3 – Invalid-Radical during reading

Due to the increase in stimulus size, we changed our criteria to instead remove trials with naming latencies above 1200 ms and below 300 ms. Trial removal rate was lower than 3%. The Parsimonious Model had the formula:

$$ > \mathrm{lmer}\_\mathrm{object}=\mathrm{lmer}(\mathrm{RT} \sim {\rm{congruence}}+(1|\mathrm{subject})+(1|\mathrm{pair})+(1|\mathrm{color}),{\rm{data}}={\rm{stroop}}\mathrm{.data})$$

Relative to the *Neutral-Control* trials, there was a Stroop interference effect for incongruent *Invalid-Radical* characters (M = 30 ms, SE = 10.3 ms, *t* (234.41) = 2.879, *uncorrected p* = 0.0044, *corrected p* = 0.0085, *d* = 0.29), the facilitation effect was not significant (M = 24 ms, SE = 13.7 ms, *t* (105.77) = 1.775, *uncorrected p* = 0.0787, *corrected p* = 0.1435). No speed-accuracy trade-off was observed, since there was no difference in error rate between different conditions. The mean accuracy of the phrase recognition task was 75%.

### Experiment 4 – Associative-Radical

The trimming criteria were the same as Experiment 1 and 2. Trial removal rate was lower than 1%. The Parsimonious Model had the formula:

$$\begin{array}{rcl} > \mathrm{lmer}\_\mathrm{object} & = & \mathrm{lmer}(\mathrm{RT} \sim {\rm{congruence}}\ast \mathrm{character}\_\mathrm{type}+(1+{\rm{congruence}}+\mathrm{character}\_\mathrm{type}|\mathrm{subject})\\  &  & +\,(1|\mathrm{pair})+(1|\mathrm{color}),{\rm{data}}={\rm{stroop}}\mathrm{.data})\end{array}$$

Relative to *Neutral-Control* trials, the Stroop interference effect was found in both the *Invalid-Radical* condition (M = 14 ms, SE = 6.5 ms, *t* (91.99) = 2.102, *uncorrected p* = 0.0383, *corrected p* = 0.0383, *d* = 0.16) and the *Associative-Radical* condition (M = 16 ms, SE = 6.6 ms, *t* (92.86) = 2.514, *uncorrected p* = 0.0137, *corrected p* = 0.0137, *d* = 0.19). There was no difference between the two interference effects (M = ~3 ms, SE = 8.8 ms, *t* (617.28) = 0.312, *uncorrected p* = 0.7549, *corrected p* = 0.7549). Analysis of error rates indicates that the speed accuracy trade-off can be ruled out, since no such effect was found in both conditions relative to *Neutral-Control* trials.

### Data Availability Statement

The datasets gathered and analyzed during the current study are available on our lab domain, http://epa.psy.ntu.edu.tw/data_repository/StroopRadicalProcessing_Data.rar.

## General Discussion

In this study, we conducted four experiments using the Stroop paradigm to examine whether there is semantic activation of the phonetic radicals in viewing Chinese compound characters. Results showed that Stroop effects were reliably obtained for Chinese characters that were color names (Experiment 1), and also for characters that were unrelated to color yet contained phonetic radicals that were color-name characters when standalone (Experiments 1 and 2). Stroop interference effects were also evident in a near-reading condition when the colored character was placed at the end of a four-character phrase (Experiment 3), and when the phonetic radicals were not color names but each had a meaning related to a color (Experiment 4). Taken together, these results provide strong evidence for the automatic and independent semantic activation of the phonetic radical, even when the function of the phonetic radical, by definition, is to cue the pronunciation rather than the meaning of the compound character; and more remarkably, even when semantic activation of the phonetic radical would eventually cause interference with that of the whole character.

Biederman and Tsao^29^ first used Chinese characters as stimulus materials in the Stroop paradigm and found the Stroop effect for Chinese characters that were color names, a robust result that has since been replicated repeatedly^30–36^. Spinks *et al*.^35^ took one step further and obtained the Stroop effect for homophones of color-name characters. Our findings of robust Stroop effects for Chinese characters that were color names and homophones of color names in Experiment 1 thus add one more piece of evidence that is consistent with previous studies.

The novel contribution of this study, however, is to extend beyond the basic findings of the Stroop effect for whole characters to that for the embedded phonetic radicals that were color names (Experiment 1 to 3), or carried a meaning of an object associated with color (Experiment 4). This effect cannot be attributed to priming from stimulus set or task set (Experiment 2 to 4) since the Stroop effect was still observed even without potential priming from exposure to trials containing color characters or homophones (i.e., *Valid-Radical* characters), from congruent trials in the *Invalid-Radical* characters, and from lexical correspondence of the same color names without necessarily involving semantic activation. Furthermore, although the number of incongruent trials is doubled compared to congruent ones, the Stroop effect should not be affected^37^; if the Stroop effect is reduced when the number in the incongruent conditions is larger or the proportion of color words is higher^38^, our results should represent an underestimation, which would not affect our conclusion. Therefore, our results suggest that the Stroop effect indeed stemmed from phonetic radical’s semantic activation (please refer to supplemental material 3.1 to an in-depth discussion on our use of the Stroop paradigm).

Shifting our focus to the subtleties in Experiments, there are a few interesting points. First, in Experiment 1, in the *Invalid-Radical* condition in which a character containing two radicals was presented, even though the Stroop effect demonstrated that the meaning of the phonetic radical can be activated, the semantic radical and the whole character’s semantic information (both are not related to any color names) should theoretically also be automatically processed, causing an interruption to the Stroop effect. This is consistent with the pattern of Experiment 1’s results, where the Stroop interference was significantly weaker in the *Invalid-Radical* condition (39 ms) than the *Color-Character* condition (79 ms). The second point is that, in the same Experiment, there was no difference in Stroop effects between the *Valid-Radical* and *Invalid-Radical* conditions. Technically, the distinction between the *Valid-* and *Invalid-Radical* conditions lies in how faithfully the phonetic radical conveys the pronunciation to the whole character. As the whole characters in *Valid-Radical* condition share the same phonologies with actual color names, we had expected a moderately higher interference effect in the *Valid-Radical* condition compared to that in the *Invalid-Radical* condition; surprisingly, there was none. We infer this lack of difference to be the consequence of there being little to no pronunciation cuing from phonetic radicals in the Stroop paradigm used here, even when there is a task demand to name the color which might have triggered the activation of pronunciation. Thirdly, in Experiment 2, we focused only on the critical (*Invalid-Radical*) condition to avoid potential confounding of priming from trials in the *Color-Character* and *Valid-Radical* conditions, the large difference found between the *Invalid-Radical* condition’s Stroop effects of Experiment 1 and 2 (73 ms vs. 44 ms when summing facilitation and interference together) showed that there might indeed be some priming from the two removed conditions. Yet, in absence of these priming effects the Stroop effect was still significant in Experiment 2, albeit smaller. Thus this strongly supports the view that phonetic radicals have been through semantic activation during each trial.

For those readers more familiar with research on Stroop effect, they might suspect that our smaller results in Experiment 2 are similar to the Stroop Dilution Effect^39^. In the Stroop dilution effect, the Stroop effect is reduced when the color word is accompanied by one or multiple neutral words, and attentional competition between these words has been the explanation for it. If we consider the semantic radical and the whole character from our Invalid-Radical condition as “neutral” characters (i.e. possessed meaning unrelated to color), then the smaller effects in the *Invalid-Radical* condition compared to the *Color-Character* condition could indeed be explained by the Stroop dilution effect. This is relevant to our Experiment 1, 2 and 4 where the target was a single colored character and the competition had to be within the colored character per se, since there was no neutral word that accompanied the colored target character. In Experiment 3, we had a different case where the colored target character was accompanied by three uncolored (black) neutral characters. However, the Stroop effect we obtained from Experiment 3 was not reduced compared to that from Experiment 2, where only one character was presented (21 ms facilitation and 23 ms inhibition in Experiment 2, and 24 ms facilitation and 30 ms inhibition in Experiment 3). This is consistent with the finding that neutral words would not dilute the Stroop effect when they were not colored^40^. However, readers should keep in mind that we are drawing a conclusion from two independent experiments in this study.

Another potential argument is that the Stroop effects found in the present study could also be explained by the neighborhood effects among whole character representations in the orthographic lexicon, rather than the effects at the radical level. We do not think orthographic neighborhood at the whole character level could explain what we observed for the following reasons: 1) In Experiment 1 in which three conditions were manipulated (*Color-Character*, *Valid-Radical*, *Invalid-Radical*), the characters from *Valid-Radical* and *Invalid-Radical* conditions have the same neighborhood size (i.e., they share the same phonetic radical and thus belong to the same group). However, our results showed that the Stroop effect was significantly different between the *Color-Character* and *Valid-Radical* conditions. Had neighborhood size effects contributed to the Stroop effects, we should have observed the same pattern of results between *Color-Character* vs. *Valid-Radical* and *Color-Character* vs. *Invalid-Radical* conditions. 2) It has been shown that Stroop effects interact with neighbor frequency^41^, and yet our results do not appear to be due to such effect, either. Regarding our stimuli in Experiment 1, characters in the *Valid-Radical* and *Invalid-Radical* conditions had high and low frequencies, respectively; but no difference in the Stroop effects was found. Had neighborhood frequency played a role, we should have observed unequal Stroop effects in these two conditions. 3) In our Experiment 4, the neighborhood sizes of the *Associative-Radical* and *Invalid-Radical* conditions were different, but their Stroop effects were still similar. 4) Most importantly, neighborhood size cannot explain the Stroop effect obtained in our *Associative-Radical* condition since it has to depend on the radical per se (e.g., the radical  “mountain” in the character  “immortal”) to trigger the association with the color, and their neighbors (e.g., ) have no association with the color at the character level.

Previous studies have examined whether the processing of radical can emerge by using the Stroop-like tasks^19,20^ and Luo *et al*.^20^ also studied whether the semantic information of phonetic radicals can be activated using a Spatial-Stroop task. Although the research questions and paradigms share similarities with ours, the current study differed from theirs in the following manipulations: 1) They did not specify their radicals as semantic or phonetic radicals (unlike the current study, where we emphasized on semantic activation of phonetic radicals), and between valid and invalid phonetic radicals. 2) They did not use a neutral condition to compare with the congruent and incongruent conditions, and thus the facilitation/interference effects cannot be assessed. Our design specifically picked well-matched neutral stimuli (matched in stroke count and usage frequency with the stimuli in the congruent and incongruent conditions) and provided more information relative to the neutral trials than simply compared congruent vs. incongruent trials. 3) They did not use foils in the stimulus sets and participants saw only two characters throughout the experiment; thus, participants may have adopted various kinds of strategies. In this study, we made efforts to avoid possible confounding factors step-by-step in a series of four experiments. Accordingly, since our designs specifically focused on the semantic activation of phonetic radicals, and have proven that even phonetic radicals can also be semantically activated and lead to the Stroop effect, we believe it is a stronger evidence that radicals are processed very much like characters.

Regarding past theories on Chinese recognition, our results add one more piece of evidence arguing against the view that character as a whole is the primary processing unit upon which reading of a text is based, and that the processing of radicals is either unnecessary or is triggered only by task demands^42–49^. According to this view, the Stroop effect should have been found only in the *Color-Character* condition (e.g., , “cyan”), but not in the *Invalid-Radical* condition (e.g., , “guess”), because the meaning of the character in the latter condition has nothing color related, as with its matched *Neutral-Control* character (, “tent”). This holistic view thus has difficulty in explaining the Stroop effects we obtained consistently in the critical *Invalid-Radical* condition from all four experiments, and even more so for the *Associative-Radical* condition in Experiment 4, since the phonetic radical itself was not even a color name.

Instead, our findings can be explained by the view that Chinese characters are recognized by activating their radicals first^6,50–57^. In this decomposition camp, most researchers have mainly focused on how radicals are processed to fulfill their semantic or phonetic functions. For example, Flores d’ Arcais *et al*.^10^ have suggested that phonetic radicals could work in the same way as sublexical letters in alphabetic words because of the over-learned orthography-phonology correspondence in reading Chinese, similar to the grapheme-to-phoneme correspondence rules in English. This view is prevalent and reflected in studies showing that the function of phonetic radicals in reading compound Chinese is to determine the phonology for the characters^9,11,12,58,59^. Similarly, previous findings also have hints of facilitation by semantic radicals on semantic processing of the characters^5,6,60,61^.

The possibility of semantic activation of the *phonetic* radicals, however, seems odd at first glance. Theoretically, maintaining that the semantic radical cues the meaning of the character, and the phonetic radical the pronunciation, is the most sensible way to represent the two kinds of radicals, provided radical function is represented. Having the meaning of the phonetic radical activated should only produce interference in parsing the meaning of the whole character, as the main function of the phonetic radical is to merely cue the pronunciation of the character. In practice, however, if the activation of the phonetic radical’s semantic information has become unavoidable, it may simply be obligatory despite the unwanted interference. The Stroop paradigm we used in this study has an advantage over other paradigms in tackling this issue: a Stroop effect indicates automatic semantic activation^17,18,62^, since the task at hand (i.e., report the font color) is irrelevant to the semantics of the characters/radicals. Therefore, the Stroop effect caused by an invalid phonetic radical, in the sense that neither its pronunciation nor its meaning provides cues to the character, reveals the indispensable nature of its semantic activation. Note that this automatic semantic activation is more in depth than mere lexical activation from recognizing the orthography of the radicals; in Lorentz *et al*.^18^, they also found Stroop effects in color associative words, suggesting that the Stroop effect is not just a product of lexical activation. This is also confirmed in our Experiment 4 where *Associative-Radical* condition also gave rise to the Stroop effect.

Could our findings be due to cognitive strategies, rather than automatic cognitive processing? We are fully aware of this potential problem and thus have taken efforts to exclude such a possibility from strategy related issues. The four experiments reported should have shown our efforts in doing so. After establishing the basic phenomenon of the Stroop effect from the *Invalid-Radical* condition, we excluded the other two conditions (the *Color-Character* and *Valid-Radical* conditions) in Experiment 2 to avoid the priming effects caused by color relevant stimuli from the other two conditions, and using four-character phrases in Experiment 3 to reduce the number of color-related characters presented. In Experiment 4, we used characters containing no color name related radicals (the Associative-Radicals). The Stroop effects were consistently found over the four experiments, indicating that our results should not be attributed to strategies carrying over to the critical condition. Moreover, from a different perspective, we used four-character phrases in Experiment 3 where the critical character was at the end of the phrase (i.e., the fourth character) and the other three characters were non-color-related characters. Under this situation, the color related stimuli in this experiment was merely 14%, but even presenting such a small proportion of color characters, robust Stroop effect was still found. Thus, we are content with the assumption that cognitive strategies for our task was a negligible contributing factor.

What we have found are consistent with a previous study that had generated some debate in the literature due to small effects and its theoretical implication. Zhou and Marslen-Wilson^13^ used a primed naming task and found that naming the targets (e.g., , [hei1], “black”) was facilitated when they were preceded by semantically related phonetic radicals embedded in compound primes (e.g., , [bo2], “parking” with phonetic radical “”, [bai2], “white”). When the order of primes and targets was reversed, RT cost was found instead. Based on this, it was inferred that semantic activation of phonetic radicals must be automatic; otherwise there should not have been inhibitory control over phonetic radical activation to avoid the hindrance of naming the compound characters (e.g., ). Together with studies showing that semantic radicals not only lead to semantic activation^5,6^ but also phonological activation^7^, and that phonetic radicals lead to phonological activation^9,11,12,63^, our results are in agreement with the view that radicals are processed the same ways characters are^13^. Note that this does not necessarily exclude the functional roles of the two kinds of radicals (i.e., semantic or phonetic), since they can be carried through a semantic or a phonological network consisting of characters that contain a given radical, in addition to the activation of its own character representation (see also^64^).

Previous studies have revealed certain similarities between morphemic processing in English and radical processing in Chinese, such as being decomposed regardless of word frequency and position^1,50,56,64,65^. This study went one step further and asked whether the radical processing in Chinese is also similar to the morphological decomposition in English whereby only semantics of transparent morphemes but not opaque morphemes are activated. For English, Rueckl and Aicher^66^ found that for most of the masked priming effects they reviewed, equivalent priming effects were obtained for semantically transparent (*teach* in *teacher*) and opaque (*corn* in *corner*) morphemes. Although they found in their own experiments a larger priming effect for semantically transparent than opaque primes when the prime and target were intervened with 7–13 trials (called long-term priming effect), there was still no priming effects for the opaque prime-target pairs compared to the control pair. On top of that, there was no semantic priming effect for semantically related pairs (e.g., water-ocean). Instead of the qualitative description of the results, Feldman *et al*.^67^ used a meta-analysis of the literature mentioned in Ruckle and Aicher^66^ and found significantly larger priming effects for the transparent primes, same as the results from their own investigation (but note that they added identical pairs as the context to facilitate the priming effect). Nevertheless, there was no priming effects for the semantically opaque prime-target pairs. In summary, although there are models that propose morphemes as access units to word recognition^68^ in which words are decomposed into morphemes before lexical access, the findings from studies on English word recognition suggest that semantics of morphemes are not necessarily activated, at least not for the semantically opaque words (e.g., *secretary* and *corner*).

When we draw the conceptual similarity between semantically opaque pairs in English and invalid radical characters in Chinese, it is intriguing that our results instead indicate that radicals in Chinese character processing undergo a lexical processing just like a character, and the semantics of the sub-character radicals are activated even though when doing so might only serve to distract semantic processing of the whole character. Hence, the unavoidable semantic activation of sub-character radicals may constitute a unique feature in Chinese character processing, which may expand our perspectives when we comprehend studies comparing Chinese with other languages (refer to supplemental material 3.2 for a brief discussion on our result’s implication on currently available reading models).

## Electronic supplementary material

Supplementary Information
