# Supplementary material for: Lexical processing of Chinese sub-character components: Semantic activation of phonetic radicals as revealed by the Stroop effect
Source: Sci Rep. 2017 Nov 17;7:15782. doi: 10.1038/s41598-017-15536-w (PMC5693949; doi:10.1038/s41598-017-15536-w)
Supplement: Supplementary file 1 — Supplementary Information [file 41598_2017_15536_MOESM1_ESM.docx]

**Supplemental Materials for:**

**Lexical processing of Chinese sub-character components:
Semantic activation of phonetic radicals as revealed by the Stroop effect**

Su-Ling Yeh^123^*, Wei-Lun Chou^4^, and Pokuan Ho^1^

^1^ Department of Psychology, National Taiwan University, Taipei, Taiwan

^2^Graduate Institute of Brain and Mind Sciences, National Taiwan University, Taipei, Taiwan

^3^Neurobiology and Cognitive Neuroscience Center, National Taiwan University, Taipei, Taiwan

^4^Department of Psychology, Fo Guang University, Yilan, Taiwan

*Corresponding Author:

Su-Ling Yeh

Department of Psychology

National Taiwan University

Taipei 10617, Taiwan

Phone: (886) 2-33663097

Fax: (886) 2-23629909

Email: [suling@ntu.edu.tw](mailto:suling@ntu.edu.tw)

**Supplemental Material 1.1**.

Chinese characters and their controls (*Neutral-Control* characters) used in this study

| Condition  (Associated Color) | Character  (Phonetic radical) | Meaning  (Phonetic radical) | Pronunciation | Frequency  (Stroke count) | Neutral-Control  (Phonetic radical) | Meaning  (Phonetic Radical) | Pronunciation | Frequency  (Stroke count) |
| --- | --- | --- | --- | --- | --- | --- | --- | --- |
| *Color-Character* | | | | | | | | |
| Cyan | 青 | cyan | [qing1] | 257 (8) | 具 | tool | [ju4] | 262 (8) |
| Yellow | 黃 | yellow | [huang2] | 513 (12) | 曾 | already | [ceng2] | 543 (12) |
| Red | 朱 | red | [zhu1] | 117 (6) | 丟 | discard | [diu1] | 117 (6) |
| *Valid-Radical* | | | | | | | | |
| Cyan | 清 (青) | clear (cyan) | [qing1] | 1760 (11) | 理 (里) | reason (length unit) | [li3] | 1666 (11) |
| Yellow | 潢 (黃) | pond (yellow) | [huang2] | 2 (15) | 諄 (享) | iterate (enjoy) | [zhun1] | 2 (15) |
| Red | 珠 (朱) | pearl (red) | [zhu1] | 44 (10) | 軒 (干) | pavilion (dry) | [xuan1] | 45 (10) |
| *Invalid-Radical* | | | | | | | | |
| Cyan | 猜 (青) | guess (cyan) | [cai1] | 130 (11) | 帳 (長) | tent (long) | [zhang4] | 130 (11) |
| Yellow | 橫 (黃) | horizontal (yellow) | [heng2] | 73 (16) | 榜^#^ (旁) | placard (side) | [bang3] | 65 (14) |
| Red | 殊 (朱) | different (red) | [shu1] | 62 (10) | 勒 (革) | strangle (leather) | [le4] | 62 (11) |
| *Associative-Radical* | | | | | | | | |
| Cyan | 仙 (山) | immortal (mountain) | [xian1] | 98 (5) | 佔 (占) | occupy (fortune telling) | [zhan4] | 94 (7) |
| Yellow | 淦 (金) | river name (gold) | [gan4] | 1 (11) | 浯 (吾) | river name (*archaic* me) | [wu2] | 1 (10) |
| Red | 恤 (血) | pity (blood) | [xu4] | 5 (9) | 怯 (去) | afraid (go) | [qie4] | 5 (8) |

* Frequency per million, based on Tsai (1996). ^#^ The *Neutral-Control* character 齊 (orderly, [qi2], frequency: 72, stroke count: 14) was used in Experiment 2 and 3.

**Supplemental Material 1.1**. (Continued)

Linguistic characteristics at the radical level

| Condition  (Associated Color) | Character  (Phonetic radical) | Consistency* | Phonetic Combinability^^^ | Semantic Combinability^&^ | Neutral-Control  (Phonetic radical) | Consistency * | Phonetic Combinability^^^ | Semantic Combinability^&^ |
| --- | --- | --- | --- | --- | --- | --- | --- | --- |
| *Color-Character* | | | | | | | | |
| Cyan | 青 | -- | -- | -- | 具 | -- | -- | -- |
| Yellow | 黃 | -- | -- | -- | 曾 | -- | -- | -- |
| Red | 朱 | -- | -- | -- | 丟 | -- | -- | -- |
| *Valid-Radical* | | | | | | | | |
| Cyan | 清 (青) | 0.50 | 16 | 226 | 理 (里) | 0.91 | 10 | 65 |
| Yellow | 潢 (黃) | 0.57 | 6 | 226 | 諄 (享) | 0.14 | 7 | 123 |
| Red | 珠 (朱) | 0.80 | 10 | 65 | 軒 (干) | 0.06 | 15 | 30 |
| *Invalid-Radical* | | | | | | | | |
| Cyan | 猜 (青) | 0.06 | 16 | 35 | 帳 (長) | 0.67 | 6 | 20 |
| Yellow | 橫 (黃) | 0.13 | 8 | 289 | 榜 (旁) | 0.54 | 10 | 161 |
| Red | 殊 (朱) | 0.20 | 10 | 17 | 勒 (革) | 0.50 | 10 | 31 |
| *Associative-Radical* | | | | | | | | |
| Cyan | 仙 (山) | 0.20 | 5 | 150 | 佔 (占) | 0.23 | 12 | 150 |
| Yellow | 淦 (金) | 0.17 | 6 | 379 | 浯 (吾) | 0.71 | 14 | 379 |
| Red | 恤 (血) | 0.50 | 4 | 146 | 怯 (去) | 0.11 | 9 | 146 |

^*^ Consistency is defined as the ratio of the number of characters sharing a phonetic radical that have the same pronunciation to the number of characters sharing that phonetic radical. Tonal differences are not taken into account. ^^^ Phonetic Combinability is defined as the number of characters that share a phonetic radical. ^&^ Semantic Combinability is defined as the number of characters that share a semantic radical. All of the data were based on Chang, Hsu, Tsai, Chen, and Lee (2016)^1^ and sinica.edu.tw database.

**Supplemental Material 1.2**.

Examples of Chinese characters and phrases used in Experiment 3

| Condition | *Invalid-Radical* character | *Neutral-Control* character |
| --- | --- | --- |
| Critical character | 猜 | 帳 |
| Meaning | guess | tent |
| Pronunciation | [cai1] | [zhang4] |
| Phonetic radical | 青 | 長 |
| Meaning | cyan | long |
| Pronunciation | [qing1] | [chang2] |
|  |  |  |
| **Stimuli Presented** |  |  |
| Phrases* | 兩小無**猜** | 請先付**帳** |
| Meaning | describes childhood innocence | pay first, please |
| Pronunciation | [liang3 xiao3  wu2 cai1] | [qing3 xian1  fu4 zhang4] |

* The bold characters in the phrases (the last character in the phrase) were colored in cyan, yellow, or red.

**Supplemental Material 1.3**

Chinese phrases used in Experiment 3

|  | Critical character | Phrases* Frequency^#^ (Stroke count) | | |  |
| --- | --- | --- | --- | --- | --- |
| *Invalid-Radical* character | | | | | |
|  | 猜 | 兩小無**猜** 467000 (34) | 隨便亂**猜** 521000 (48) | 不要瞎**猜** 71900 (39) |  |
|  | 橫 | 阡陌縱**橫** 98300 (46) | 老淚縱**橫** 519000 (50) | 跋扈專**橫** 39800 (50) |  |
|  | 殊 | 身分特**殊** 63600 (31) | 相差懸**殊** 462000 (49) | 待遇特**殊** 78100 (41) |  |
| *Neutral-Control* character | | | | | |
|  | 帳 | 請先付**帳** 39800 (37) | 銀行轉**帳** 450000 (49) | 記流水**帳** 63300 (34) |  |
|  | 齊 | 壽與天**齊** 218000 (46) | 非常整**齊** 341000 (49) | 排放整**齊** 76400 (49) |  |
|  | 勒 | 海倫凱**勒** 131000 (43) | 褲帶緊**勒** 12100 (51) | 像個彌**勒** 122000 (52) |  |

* The bold characters in the phrases were colored in cyan, yellow, or red, while the other three characters were in black.

^#^ Unlike single characters, we did not come across a reliable source that documents the frequencies of these phrases; hence we utilized the Google Search Engine for these numbers as a last resort. However, due to the rapid expansion of online information, readers should expect these numbers to change over time.

### **Supplemental Material 2 - R Implementation**

### **Brief Notes**

- Since we had carefully matched our stimuli such that each item had a corresponding control word with similar usage frequency and stroke count, we assigned the same pair number to each matched pair as a way of keeping them “yoked” in the model.
- For ‘pair’, we consider the same characters presented in different colors different stimuli, as the response task is color naming.
- We have averaged the results from repeated trials in different blocks.

### **The Three Steps**

As stated in the main manuscript, our analysis can be divided into 3 steps:

1. Determining the ‘most-maximal-possible-model’ using ‘lme4’.
2. Reduce the found model systematically to avoid over-specification using ‘RePsychLing’.
3. Construct comparison tables using ‘lsmeans’ with the final model from step 2 as a parameter.

We will use our Experiment 1 as an example to explain the steps.

### **Determining the ‘Most-Maximal-Possible-Model’**

We first attempt to generate the ‘Maximal-Model’ with the following formula:

> max_model = lmer(RT ~ congruence * character_type +
(1 + congruence + character_type + congruence:character_type | subject) +
(1 + congruence + character_type + congruence:character_type | pair) +
(1 + congruence + character_type + congruence:character_type | color),
data = stroop.data)

“RT” is the dependent variable. “Character type” and “Congruence” are our assignments of fixed factors. (1+…|subject) + (1+…|pair) + (1+…|color) represents our selection of random factors. This formula essentially tells the “lmer()” function from ‘lme4’ to generate a model that maps the relationship between the dependent variable (i.e., RT) and the fixed factors (i.e., “Character type” and “Congruence”) while isolating random variations from random factors (i.e., “subjects”, “item pairs”, and “color conditions”). The random structure shown here is the maximal one, which means “not only will the model assume a different RT intercept, it will assume effects from fixed factors, as well as their interactions, vary for each data point from the random factor.” The “1” inside each random factor is referred to as the “random intercept”, and terms after the random intercept are called “random slopes”.

In practice, the ‘Maximal-Model’ is sometimes too complex for the dataset and may cause convergence errors or warnings. When this happens, we must reduce our model formula; and the one that converges successfully with the most elaborate random effect structure possible is what we will coin the ‘Most-Maximal-Possible-Model’ (MMP-Model).

To find the MMP-Model, we first attempt to construct a ‘Maximal-Model’. If it converges successfully, then it is also the MMP-Model; if not, we reduce its random effect structure one random slope at a time until convergence. Our order of removal is as follows: a) the higher order interactions are removed first. b) with our assumption that there would be more between-subject variation, followed by that of item-pair, then color; we would remove random slopes from random factors in the following order: color, item-pair, subject. c) Because our planned comparisons primarily focus on the differences between Congruence conditions, we would remove ‘Character Type’ first, followed by ‘Congruence’.

Once we have determined the MMP-Model, we can move on to the next step.

For our Experiment 1, the MMP-Model is:

> max_model = lmer(RT ~ congruence * character_type +
(1 + congruence | subject) + (1 | pair) + (1 | color), data = stroop.data)

### **Finding the ‘Parsimonious Model’**

According to Bates^2^, we should also assess the dimensionality assumed in the MMP-Model using the ‘rePCA()’ function from the ‘RePsychLing’ package. Essentially, this checks for any components in the random effect structure that do not contribute to the model fitting, which means they could be removed without hurting the MMP-Model’s goodness-of-fit. Then a model without those components (i.e. a ‘Reduced’ model) would be constructed, assessed with the ‘rePCA()’ function, and compared with the MMP-Model using Likelihood-Ratio Test to decide if the reduced model differs in goodness-of-fit (we use *p* = .1 as the cut-off value). If not, it implies the MMP-Model’s random effect structure might have been over-specified.

This is usually an iterative process where finding the optimal model requires comparisons between numerous models at several complexities. However, as even our full-fledged random effect structure may not be that complicated to begin with, we limited ourselves to only one iteration in our procedure.

Because this step can be non-linear and difficult to explain in words, we encourage the readers to refer to our online data repository where we also offer line-by-line explanations of our R commands.

For our Experiment 1, because the reduced model did not differ substantial enough from the MMP-Model in goodness-of-fit, we will use the reduced model’s formula to conduct later analyses.

### **Construct Comparison Tables**

We first construct the linear mixed effect model with the final formula from the last step.

> lmer_object = lmer(RT ~ congruence * character_type +
(1 | subject) + (1 | pair) + (1 | color), data = stroop.data)

Then the comparison table using the model is built with the following commands:

> lsm_object = lsmeans(lmer_object, list(pairwise ~ congruence|character_type))

> summary(contrast(lsm_object, "trt.vs.ctrl", ref = c(2)), by = NULL, adjust = "none")

“trt.vs.ctrl” compares each condition with the designated reference condition. “ref = c(2)” points the reference condition to *Neutral-Control* condition. “by = NULL” shortens lsmeans output.

“adjust = ‘none’” displays unaltered p-values.

To correct for multiple comparisons, we remove the ‘by’ and ‘adjust’ parameters:

> summary(contrast(lsm_object, "trt.vs.ctrl", ref = c(2)

This will give us corrected p-values inside each character type.

And for testing the contrasts of Stroop effects across character types:

> contrast(lsm_object, interaction = c("pairwise"))

“pairwise” lists out all possible pairwise contrasts with their respective statistics. We will apply our Holm-Bonferroni correction manually.

**Effect Size Calculation**

Here we adopt Cohen’s *d* as our measure for effect sizes. The ‘lsmeans’ package does not provide the standard deviation for the contrasts nor offers a way to obtain the effect size, so we estimated the standard deviations based on the given standard errors from ‘lsmeans’ output and the number of observations, which takes both the number of subjects and items into account.

For example, to get the facilitation effect size from the comparison of *Color-Character* condition in Experiment 1, the number of observations is (1 item × 3 color × 28 subjects) which gives us 84 total observations (N = 84) for ‘Congruent’ condition. The number for *Neutral-Control* trials is the same as the items were paired based on frequency and stroke count.

**Supplemental Material 3**

**3.1 Stroop Paradigm Discussion:**

The use of the Stroop paradigm in context of embedded radicals might give rise to a few possible arguments: one stimulus-related, and three task-related. First, since Stroop experiments are inherently constrained by the small number of stimuli that fit the stringent experimental criteria, it results in a potential scenario in which the effects are also caused by unique properties of certain characters selected as stimuli other than their semantic relations to color names. To address this issue which essentially stems from between-item variations, we used the LME Modeling^3^ that treated word items as a random factor. This analysis could effectively minimize the possibility that the effects we observed were due to an anomaly effect. The Stroop effects we found across the four experiments should thus be free from this problem.

Second, a task-related argument is that single-character trials might encourage the decomposition of characters into radicals, compared to when characters are embedded in sentences^4^. Being aware of this, we used four-character phrases as stimuli in Experiment 3 to provide a better approximation to normal reading situations which should discourage such decomposition. The presence of Stroop interference from *Invalid-Radical* characters embedded in phrases thus confirms that the effect cannot be solely explained by the single-character decomposition proposition.

Third, another concern is that the color-naming task *per se* could have resulted in semantic activation of the phonetic radical that is a color name, regardless of its congruence with the color it is presented in. For example, 青 (cyan, [qing1]) in 猜 (guess, [cai1]) could have been semantically activated simply because it is a color name, even if the color it appears in is not congruent. Since this concern relies on the precondition that the phonetic radical has to be a color name, it is easily testable using items that are not color names but are nonetheless related to color; which we investigated in Experiment 4. Our result that *Associative-Radical* condition yielded the same level of Stroop interference as *Invalid-Radical* condition is indicative that this effect is not attributable to mere task demand because the naming task correlates little with the *Associative-Radical* characters and their respective phonetic radicals.

One might then push on to the extreme and ask whether it is possible that pre-activation of color semantics is also a necessary condition for accessing *Associative-Radical* through this indirect connection. For example, prior research found that the word *capable* could prime categorization of the target word *radish* as a vegetable through an indirect connection via the recognition of *cabbage*; however, this priming effect vanished when the task was changed into a two-alternative forced-choice discrimination task^5^. If our effect depends on the color-naming task to provide the pre-activation of color semantics, the question whether it can still be considered as automatic semantic activation necessarily follows. Note that by “automatic”, we adopt the commonly held criteria of without intention and high resistance to suppression^6,7^. Since the meaning of the phonetic radicals would directly hinder the semantic processing of the whole characters they are part of, such a semantic activation must be automatic. Whether the Stroop paradigm is a pre-requisite for this kind of automaticity requires further investigation using even more indirect measures than the color-naming Stroop task to see whether the effect persists without any kinds of task demand.

**3.2 Implications on Current Linguistic Models:**

Our findings of semantic activation of phonetic radicals may be explained by Perfetti et al.’s *Lexical Constituency* Model^8^ after some modifications, but not by their earlier *Interactive* *Constituency* Model^9^. In the Interactive Constituency Model^9^, word identification entails retrieval of the orthography, phonology, and semantics of a word, with each constituent being part of the identification process. Visual inputs go through the stroke-analysis level, and then feed into three subsystems: the character orthographic subsystem, the non-character orthographic subsystem, and the character phonological subsystem; and all three subsystems can connect to the meaning subsystem. If the phonetic and semantic radicals can be characters themselves, they should activate the character orthographic subsystem and connect to the phonological subsystem and the meaning subsystem. Otherwise, they should activate the non-character orthographic (radical) subsystem. However, separating the character orthographic subsystem from the non-character radical subsystem requires an initial lexical decision; that is, knowing whether the radicals can be standalone characters or not, and this unavoidably invites the criticism of circular reasoning. Perfetti et al.’s Lexical Constituency Model^8^, on the other hand, aims at developing a general model of reading (the role of radicals is thus similar to the role of sublexical letter clusters in English) and removing the distinction of the two orthographic subsystems in their earlier model. In this model, radicals and their relative positions serve as the graphic units. With the combination of radical inputs to one of four possible structures (horizontal, vertical, partially enclosed, and completely enclosed; see also ^10^), the orthographic representation of the character is activated; which, in turn, activates its corresponding phonology and semantics. However, the Lexical Constituency Model may have underestimated the radicals’ role in this semantic activation since our finding has shown that radicals are semantically activated, even if their meanings conflict with that of the whole characters they are embedded in. In order to explain the semantic activation of phonetic radicals found in our experiment, the Lexical Constituency Model should be modified in a way that allows radicals that can also be standalone characters to act like characters and would connect to their respective phonology and semantics.

Additionally, our results may also be partially explained by the *Multi-Level Interactive-Activation* Model^11-15^, which also includes the three necessary components (orthography, phonology, and semantics) for reading, albeit quite distinct from the *Lexical Constituency Model*. In the orthographic subsystem, there are multiple levels of representation, including strokes, radicals, characters, and words. Character recognition is mediated by activation of position-sensitive radical units, which, in turn, are activated through their correspondent stroke-level representations and the position-free radical representations. This model assumes that compound characters, as well as radicals as standalone characters, connect to their corresponding phonological and semantic representations, and thus can explain why phonetic radicals which can be characters by themselves also lead to semantic activation of their own. However, the model’s assumption that pronunciation of the character can only be accessed through the mediation of a concept unit called *lemma* may be too narrow when our data is taken into account. This strictly unidirectional connection from meaning to phonology precludes the possibility of phonology-mediated semantic activation; that is, with reference to our experiment, the possibility to phonologically activate color names by phonetic radicals in our valid-radical condition. However, if we exclude such possibility, then we should expect to see a relatively equal Stroop interference effect (facilitation effect has been known to be less consistent and thus is not discussed here^16^) between *Valid-Radical* and *Invalid-Radical* condition in Experiment 1, as the only difference between them is in whether the pronunciation of the character matches the phonology of a color name. Thus, the trend that the *Valid-Radical* condition yielded higher Stroop interference effect than the *Invalid-Radical* condition may imply that the pronunciation of the character played a role in facilitating semantic activation. Regardless, since the bulk of our experiments focused heavily on the *Invalid-Radical* condition, we invite future studies to scrutinize the contribution of character phonology on semantic processing.

# **References**

1 Chang, Y.-N., Hsu, C.-H., Tsai, J.-L., Chen, C.-L. & Lee, C.-Y. A psycholinguistic database for traditional Chinese character naming. *Behavior Research Methods* **48**, 112-122, doi:10.3758/s13428-014-0559-7 (2016).

2 Bates, D., Kliegl, R., Vasishth, S. & Baayen, H. Parsimonious mixed models. *arXiv preprint arXiv:1506.04967* (2015).

3 Baayen, R. H., Davidson, D. J. & Bates, D. M. Mixed-effects modeling with crossed random effects for subjects and items. *J Mem Lang* **59**, 390-412, doi:10.1016/j.jml.2007.12.005 (2008).

4 Chen, H. C. Character detection in reading Chinese: Effects of context and display format. *Chinese Journal of Psychology* **26**, 29-34 (1984).

5 Bell, D., Forster, K. & Drake, S. Early semantic activation in a semantic categorization task with masked primes: Cascaded or not? *J Mem Lang* **85**, 1-14, doi:10.1016/j.jml.2015.06.007 (2015).

6 Jonides, J. Voluntary versus automatic control over the mind’s eye’s movement. *Attention and performance IX* **9**, 187-203 (1981).

7 Moors, A. & De Houwer, J. Automaticity: a theoretical and conceptual analysis. *Psychol Bull* **132**, 297-326, doi:10.1037/0033-2909.132.2.297 (2006).

8 Perfetti, C. A., Liu, Y. & Tan, L. H. The lexical constituency model: some implications of research on Chinese for general theories of reading. *Psychol Rev* **112**, 43-59, doi:10.1037/0033-295X.112.1.43 (2005).

9 Perfetti, C. A. & Tan, L. H. The constituency model of Chinese word identification. *Reading Chinese script: A cognitive analysis*, 115-134 (1999).

10 Yeh, S. L. & Li, J. L. Role of structure and component in judgments of visual similarity of Chinese characters. *J Exp Psychol Hum Percept Perform* **28**, 933-947, doi:10.1037//0096-1523.28.4.933 (2002).

11 Ding, G., Taft, M. & Zhu, X. The representation of radicals that can be used as characters. *Acta Psychologica Sinica* **32**, 21-26 (2000).

12 Taft, M. Processing of characters by native Chinese reader. *Handbook of East Asian Psycholinguistics, Vol. 1: Chinese*, 237-249 (2006).

13 Taft, M., Zhu, X. P. & Peng, D. L. Positional specificity of radicals in Chinese character recognition. *J Mem Lang* **40**, 498-519, doi:DOI 10.1006/jmla.1998.2625 (1999).

14 Taft, M. & Zhu, X. P. Submorphemic processing in reading Chinese. *J Exp Psychol Learn* **23**, 761-775, doi:Doi 10.1037//0278-7393.23.3.761 (1997).

15 Taft, M., Zhu, X. & Ding, G. The relationship between character and radical representation in Chinese. *Acta Psychologica Sinica* **32**, 1-12 (2000).

16 MacLeod, C. M. Half a century of research on the Stroop effect: an integrative review. *Psychol Bull* **109**, 163-203 (1991).
